# The Performance Characterization and Optimization of Fiber-Optic Acoustic Pressure Sensors Based on the MOEMS Sensitized Structure

**DOI:** 10.3390/s23198300

**Published:** 2023-10-07

**Authors:** Ruling Zhou, Chenggang Guan, Hui Lv, Shasha Li, Puchu Lv, Haixin Qin, Wenxiu Chu, Yikai Zhou, Yihao Zhang, Xiaoqiang Li

**Affiliations:** 1 Laboratory of Optoelectronics and Sensor (OES Lab), Hubei University of Technology, Wuhan 430068, China; zhouruling@oeslab.com.cn (R.Z.); lv@oeslab.com.cn (P.L.); chuwenxiu@oeslab.com.cn (W.C.);; 2 AOV Energy LLC, Wuhan 430068, China; 3 School of Science, Hubei University of Technology, Wuhan 430068, China

**Keywords:** EFPI fiber-optic acoustic pressure sensor, acoustic pressure-sensitive film, MOEMS, real-time coupled acoustic test

## Abstract

In order to investigate the factors affecting the acoustic performance of the extrinsic Fabry–Perot interferometer (EFPI) fiber-optic acoustic pressure sensor and to effectively improve its detection capability, this paper enhances the sensor’s detection sensitivity by adding more sensitized rings to its acoustic pressure-sensitive film. Furthermore, a novel real-time coupled acoustic test method is proposed to simultaneously monitor the changes in the spectral and acoustic metrics of the sensor to characterize its overall performance. Finally, an EFPI-type fiber-optic acoustic pressure sensor was developed based on the Micro-Optical Electro-Mechanical System (MOEMS). The acoustic tests indicate that the optimized fiber-optic acoustic pressure sensor has a sensitivity as high as 2253.2 mV/Pa, and the acoustic overload point (AOP) and signal-to-noise ratios (SNRs) can reach 108.85 dB SPL and 79.22 dB, respectively. These results show that the sensor produced through performance characterization experiments and subsequent optimization has a very high acoustic performance index, which provides a scientific theoretical basis for improving the overall performance of the sensor and will have broad application prospects in the field of acoustic detection.

## 1. Introduction

Fiber-optic acoustic sensing technology was rapidly developed in the 1970s [1]. Compared to traditional electroacoustic sensors, fiber-optic acoustic pressure sensors have the characteristics of small size, passivity, and resistance to electromagnetic interference [2]. Fiber-optic sensor technology and Micro-Optical Electro-Mechanical System (MOEMS) technology combined with the preparation of extrinsic Fabry–Perot interferometer (EFPI)-type fiber-optic acoustic pressure sensors can achieve the effective detection of extremely weak acoustic pressure signals [3]. In recent years, MOEMS-based fiber-optic acoustic pressure sensors have found a wide range of applications in national defense security, industrial non-destructive testing, and medical diagnosis [4]. However, existing acoustic pressure sensors still suffer from the disadvantages of low sensitivity, high self-noise, a low upper limit of measurable acoustic pressure, and low stability [5], which severely limit their high-quality acoustic detection in extreme environments. Thus, sensors are further expected to have high-sensitivity and acoustic overload points, as well as to generate less noise during operation.

On the other hand, the acoustic performance indexes of EFPI-type fiber-optic acoustic pressure sensors are related to the variation in the interferometric light intensity spectra generated by their F-P cavities [6], but there are only theoretical studies in this field and no empirical measurements to prove the relationship between the two. Therefore, a technical method is urgently needed to investigate the main factors affecting the acoustic performance of optical fiber sound pressure sensors and to prepare sensors with higher performance based on the existing research.

This study is based on the Passive Bidirectional Audio-Over-Fiber Transmission System (PB-AOF) [7]; the designed MOEMS chip with different sensitized ripple structures is utilized to verify that the sensitivity of sound pressure detection and other acoustic metrics can be improved, so that the final prepared sensors have the capability to detect weak acoustic signals in extreme environments. A new method of real-time coupled acoustic testing is proposed, which combines the interference spectra of the tuned F-P cavity with the simultaneous acoustic performance test to deeply analyze the influence of the parameter changes inputted to the sensor’s F-P cavity on the spectra. Then, we explore its influence on the performance of the whole acoustic system, so as to lay a good foundation for the preparation of high-performance sensors and the optimization of their performance. The final batch of fabricated EFPI fiber-optic acoustic pressure sensors with small size, low cost, high sensitivity, and high AOP has a high degree of adaptability to this system, which achieves the high-quality detection of the entire optical network architecture of long-distance audio transmission. Moreover, the fabricated MOEMS-based acoustic pressure sensor also shows the advantages of reliable consistency and low transmission loss, and it can be integrated into the multiplexing fiber-optic sensing scheme to achieve audio signal transmission in a multi-channel array [8].

## 2. Experimental Principle and Structure

The basic principle of an EFPI-type fiber-optic acoustic pressure sensor is to use light as the transmission medium of acoustic wave information, detect the small deformation of the sensitive structure caused by the acoustic wave signal with light, and then restore the acoustic wave signal into an electrical signal through optoelectronic conversion to realize the function of acoustic wave sensing [9]. It includes the three links of acoustic wave to diaphragm mechanical vibration conversion, diaphragm mechanical vibration to interference light intensity conversion, and optical signal to electrical signal conversion. The current EFPI-type fiber-optic acoustic pressure sensor mainly uses various types of diaphragm inner surfaces and fiber-optic end faces to form the two reflective surfaces of the F-P interferometer [10]. When the external acoustic pressure signal acts on the diaphragm, resulting in a small deformation, which leads to changes in the length of the F-P cavity, which in turn causes a change in the interferometric spectra.

**Figure 1:**
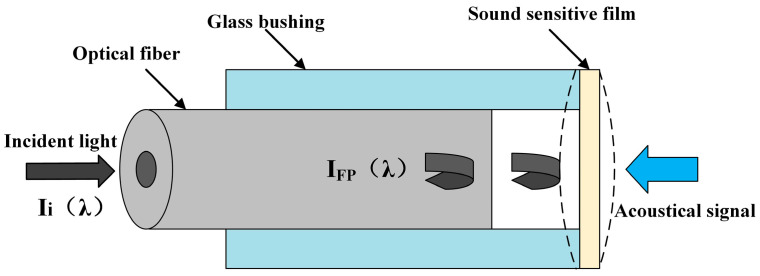
Two-beam interference model.

In this paper, the end face of the optical fiber and the inner surface of the chip of the acoustic pressure-sensitive structure are used as double reflective surfaces to form the F-P interference cavity. According to the double-beam interference theory, the theoretical model for which is shown in Figure 1, and for the EFPI-type acoustic pressure sensor considered in this paper, the F-P cavity interference light intensity spectrum can be expressed as Equation (1):

(1) $$\frac{I_{FP}\left(\lambda \right)}{I_{i}\left(\lambda \right)}\approx R_{Fiber}\left(\lambda \right)+R_{MEMS}\left(\lambda \right)+2\sqrt{R_{Fiber}\left(\lambda \right)R_{MEMS}\left(\lambda \right)}\cos\left(\frac{4\pi L}{\lambda }\right)$$

where $R_{Fiber}\left(\lambda \right)$ is the reflectivity of the end face of the optical fiber, $R_{MEMS}\left(\lambda \right)$ is the effective reflectivity of the MEMS acoustic pressure-sensitive films, $I_{i}\left(\lambda \right)$ is the total incident light intensity of the F-P cavity, $I_{FP}\left(\lambda \right)$ is the light intensity reflected by the acoustic pressure-sensitive films and then coupled into the optical fiber, L is the cavity length of the F-P cavity, and λ is the wavelength of the incident light of the light source.

From the interferometric light intensity spectrum formula, it can be seen that, under the condition that the reflectivity of the fiber end face $R_{Fiber}\left(\lambda \right)$ and the effective reflectivity of the MEMS acoustic pressure-sensitive films $R_{MEMS}\left(\lambda \right)$ are determined, the magnitude of the interferometric light intensity of the photodetectors involved in demodulating the input to the system $I_{FP}\left(\lambda \right)$ is only related to the cavity length of the F-P cavity L, the wavelength of the incident light source λ, and the incident light power of the light source $I_{i}\left(\lambda \right)$. Therefore, the control variable method is used to analyze and characterize the performance of the fiber-optic acoustic pressure sensor, and the spectral and acoustic performance of the F-P cavity are coordinated to determine the performance of the final sensor.

From Equation (2), when the effective reflectance $R_{MEMS}\left(\lambda \right)$ of the MEMS chip is equal to the reflectance $R_{Fiber}\left(\lambda \right)$ of the optical fiber end face, the interferometric light intensity spectral contrast of the output signal of the sensor is the best [11]. Then, using the loss coefficient of the F-P cavity L Equation (3) calculation, the theoretically optimal initial cavity length of the F-P cavity $L_{opt}$ is about 100 μm, where $n_{0}$ is the refractive index of the air cavity medium taken as 1.0, the $\omega_{0}$ single-mode fiber Gaussian beam mode radius is 4.9 μm, and the λ incident wavelength is 1550.12 nm. Finally, the FSR of the F-P cavity when it is at the optimal initial cavity length is calculated to be about 12 nm by using Equation (4).

(2) $$R_{MEMS}\left(\lambda \right)=R_{Fiber}\left(\lambda \right)=\varepsilon R$$

(3) $$\varepsilon =\frac{4\left[1+{\left(\frac{2\lambda L_{opt}}{\pi n_{0}\omega_{0}{}^{2}}\right)}^{2}\right]}{{\left[2+{\left(\frac{2\pi L_{opt}}{\pi n_{0}\omega_{0}{}^{2}}\right)}^{2}\right]}^{2}}$$

(4) $$FSR=\Delta \lambda =\lambda_{1}-\lambda_{2}=\frac{\lambda_{1}\lambda_{2}}{2L_{opt}}\approx \frac{\lambda_{0}{}^{2}}{2L_{opt}}$$

**Figure 2:**
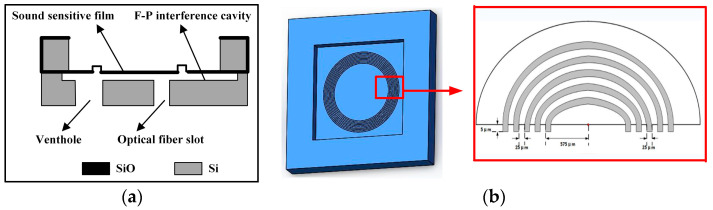
(**a**) Schematic diagram of the acoustic pressure-sensitive structure. (**b**) Schematic diagram of a cross-section of the structure of the MEMS acoustic pressure-sensitive chip.

In this experiment, the MOEMS chip is fabricated using bulk-silicon micromachining technology, which uses reactive ion etching (RIE) technology and deep reactive ion etching (DRIE) technology to design the optical F-P chamber and the core acoustic pressure-sensitive structure film of the sensor [12]. Finally, wafer-level bonding technology is utilized to realize the high-precision alignment of the two aligned integrations, which greatly improves the consistency of the sensor. As shown in Figure 2a, the etched silicon forms ventilation holes to meet the air pressure balance between the two sides of the acoustic pressure-sensitive film. The design of the fiber-optic grooves can achieve self-alignment and limitation, and the fiber-optic inserts can be directly inserted to reduce the errors caused by the manually produced encapsulated sensors and improve the efficiency of mass production.

Silicon oxide thin films compatible with the MOEMS micromachining process were chosen as the sensitive material because of its advantages of high hardness, corrosion resistance, strong structure, and high stability, and its thickness can be reduced to a nanometer scale through silicon fabrication processes. These characteristics make the silicon oxide film, as a vibrating film, have a high performance in pressure sensing [13].

The acoustic pressure-sensitive film on the surface of the chip is the core structure that determines the sensitivity of the sensor. Under the same acoustic pressure, the different designs of the sensitive film structure will result in a different range of changes in the F-P cavity, so the acoustic pressure-sensitive film design will largely affect the key indicators of the acoustic performance of the sensor. However, conventionally prepared planar silicon oxide films are prepared with residual stresses within the film that are not favorable for applications, and according to the theory of film stress analysis [14,15], when the film is thinner than the micrometer scale, due to the stress enhancement effect the residual stresses will greatly affect the sensitivity of the film [16]. Therefore, how to prepare high-performance acoustic pressure-sensitive films is a major challenge.

In this work, we design a periodic ring corrugation structure to release the initial stress of the silicon oxide film, as shown in Figure 2b, so that the center film can have a larger displacement under the same acoustic pressure to significantly improve its acoustic detection sensitivity [17]. Through specific experiments, it is verified that increasing the number of corrugated rings can effectively release the stress and reduce the rigidity of the film, thus improving the overall performance of the acoustic pressure sensor.

**Figure 3:**
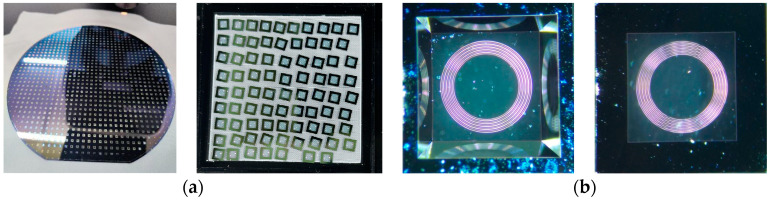
(**a**) Sample of MOEMS acoustic pressure-sensitive chip. (**b**) Microscopic observation of the front and back of the 9-ring acoustic structure chip.

The chip with the sensitized corrugated structure has a self-reflectivity R of about 21%, a size of 3.0 mm × 3.0 mm, and a thickness of 0.4 mm, in which the acoustic pressure-sensitive film has a thickness of 400 nm and a size of 2.5 mm × 2.5 mm. Figure 3a shows the physical picture of the chip with the 9-ring sensitized structure, and Figure 3b shows the microscopic observation of its front and back sides. The transmission medium of the optical signal is a single-mode fiber (SMF) with a mode field radius of 4.9 μm, and the reflectivity of the end face of the SMF can be calculated to be about 3.6% according to the Fresnel reflection principle [18]. Since the coupling efficiency of a single-mode fiber in an arbitrary harmonic field is related to the complex overlap integrals between the fiber modes and the harmonic field, $R_{MEMS}\left(\lambda \right)$ is actually slightly smaller than $R_{Fiber}\left(\lambda \right)$, and $R_{MEMS}\left(\lambda \right)$ is taken to be about 4% in this paper.

In this study, we design real-time coupled acoustic test experiments to investigate the specific factors affecting the performance of fiber-optic acoustic pressure sensors. These include the interferometric spectra of the F-P cavity, the FSR, and the interferometric intensity spectral contrast, and the various acoustic metrics of the simulated audio signal including sensitivity, systematic noise floor, AOP, and SNR. It is verified that optimizing the structure of the acoustic chip can improve the acoustic detection sensitivity of the whole sensor. Then, we use the optimized chip to adjust the cavity length and incident light wavelength of the experiment, while observing the change rule of the spectral and acoustic indicators and obtaining the best input parameters applicable to the system. Finally, based on the experimental laws and the results obtained, the batch production of a high-sensitivity and high-AOP fiber-optic acoustic pressure sensor is applicable to a passive bidirectional audio transmission system, overcoming the existing acoustic pressure sensors’ problems of low sensitivity, poor consistency, and high transmission loss.

## 3. Experimental Characteristics and Results

**Figure 4:**
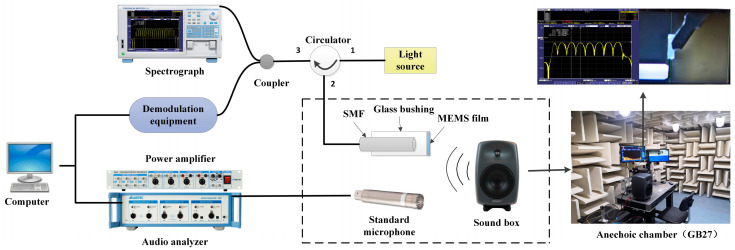
Device diagram of the experimental system.

The setup of the real-time coupled acoustic test experimental system is shown in Figure 4; to avoid external noise interference, a condenser microphone (ABTEC, AX-MC01) with a sensitivity of 45 mV/Pa was used as a reference throughout the process in a semi-anechoic chamber (ABTEC, GB27) environment. The F-P cavity coupling system and the reference condenser microphone were kept at the same distance from the loudspeaker (GENELEC, SAM8040), and were all symmetrically located along the center axis of the loudspeaker to ensure consistent sound pressure measurements. The sound source was set up by an audio analyzer to emit a standard sinusoidal signal with a frequency of 1 kHz [19]. An amplified spontaneous emission (ASE) source with a center wavelength of 1550.12 nm and a spectral width of 40 nm was used as the light source, and the light was injected into the F-P cavity coupling system via a circulator. In order to minimize the potential impact of changes in environmental factors on the sensor’s performance, the ambient humidity was controlled between 40% and 60%, and the sensor and standard microphone were placed on a heated platform (MTI-3040) with the temperature set at a constant level throughout the test. In addition, the system used a three-wavelength adaptive intensity demodulation algorithm, which had a better demodulation effect under the drastic temperature change environment, and could effectively minimize the influence of the sensor working point drift caused by the temperature change.

**Figure 5:**
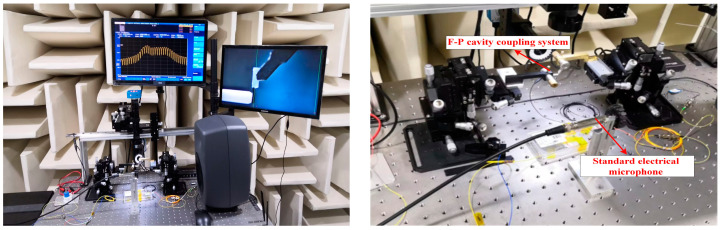
The actual experimental environment.

The spectral analyzer and the photoelectric detection and demodulation device received the optical signal reflected from the F-P cavity coupling system through another port of the circulator, where the spectral analyzer displayed the dynamic change in the F-P cavity interference light intensity spectrum [20]. The FPGA photoelectric detection and demodulation device input the interferometric light signals into the audio analyzer after converting them into voltage signals through intensity demodulation [21]. This realized the real-time synchronous monitoring of the interfering light intensity spectral changes and acoustic performance index changes; Figure 5 shows the actual experimental environment.

### 3.1. Performance Characterization

#### 3.1.1. Influence of the Number of Sensitized Rings

**Figure 6:**
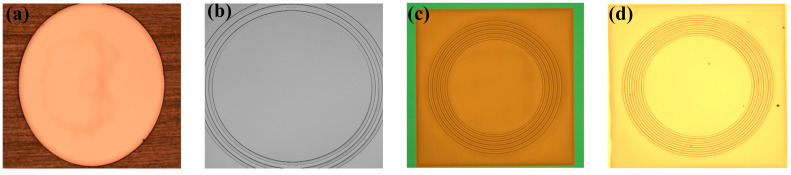
(**a**) No sensitized ripple structure chip. (**b**) 3-ring sensitized ripple structure chip. (**c**) 5-ring sensitized ripple structure chip. (**d**) 9-ring sensitized ripple structure chip.

**Figure 7:**
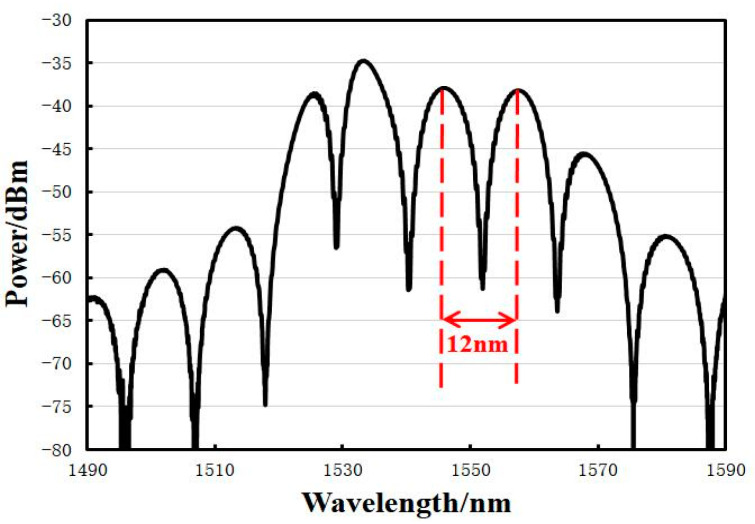
Reflectance spectrum of F-P cavity.

In order to verify that increasing the number of sensitized rings of the acoustic thin film can effectively improve the performance of the sensors, an F-P cavity system consisting of a chip with the same process parameters but containing different numbers of sensitized rings, as shown in Figure 6, and a single-mode fiber were used for testing. With the above experimental material reflectivity information and calculation formulas, the cavity lengths were adjusted as much as possible to be at the theoretically optimal initial cavity length, and the interferometric intensity spectral contrasts were all around 23 dB, as shown in Figure 7. The input optical power of the light source was adjusted to 1.5 mW, when the output sound signal was the clearest. The final results of the experiments, averaged after several sets of experiments, are shown in Table 1, which shows that, as the number of sensitized rings increases, the interfering light intensity output from the sound pressure sensor increases continuously. This results in an increase in the final voltage input to the audio analyzer, an increase in the measured voltage sensitivity of the sensor, and an increase in the bottom noise, SNR, and AOP of the whole system.

In summary, in order to release the initial stress of the film, we designed a periodic ring-type corrugated sensitizing structure on the surface of the film and verified that increasing the number of sensitized rings not only effectively improves the sensitivity of the sensor, but also optimizes other types of acoustic indices. Among them, the chip with a 9-ring sensitized ripple structure had the highest degree of adaptability to this system, making it possible to transmit audio signals over long distances while achieving the indicators of good sound quality and high audio recognition.

#### 3.1.2. Influence of F-P Cavity Length

In order to investigate the spectral and acoustic indicators of the sensor with the F-P cavity length change rule, the light source incident wavelength of 1550.12 nm and the light source of the incident light power of 1.5 mW were left unchanged. 

**Figure 8:**
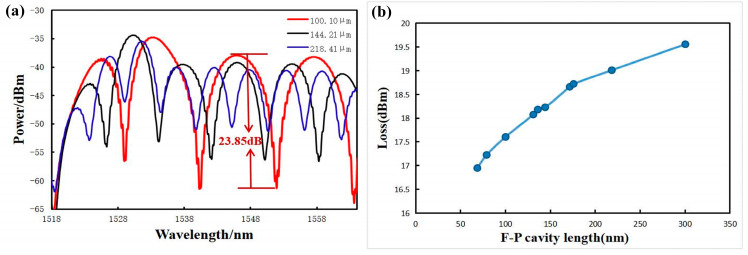
(**a**) Partial reflectance spectra of the F-P cavity; (**b**) Variation of optical loss vs. cavity length of the F-P cavity.

The different lengths of the F-P cavity under the part of the reflectance spectral map and the cavity of optical loss with the length of the change rule are shown in Figure 8. The optical loss of the whole cavity increases with the increase in F-P cavity length, and the FSR and the contrast of the interferometric light intensity spectrum in the interference spectrum decrease, which is in line with the theoretical results.

**Figure 9:**
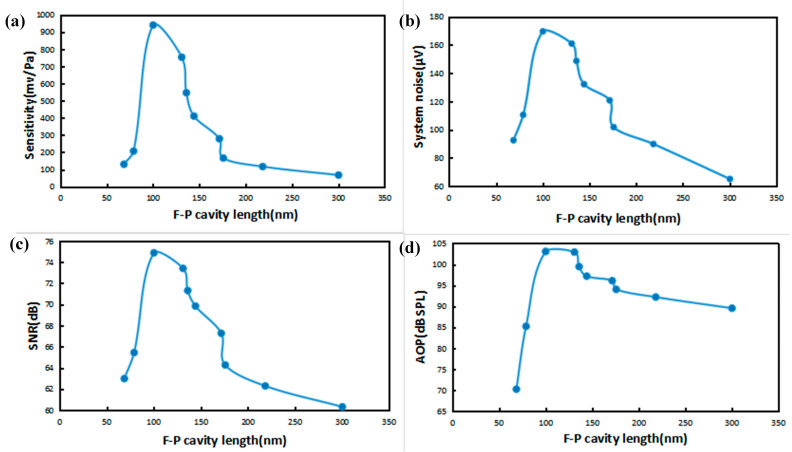
(**a**) Plot of sensitivity vs. cavity length. (**b**) Plot of system bottom noise vs. cavity length. (**c**) Plot of SNR vs. cavity length. (**d**) Plot of AOP vs. cavity length.

The four types of acoustic indicators measured with the F-P cavity length change rule are shown in Figure 9. After the optimal initial cavity length, and with the increase in F-P cavity length, the acoustic performance of the sensor gradually deteriorated, and only achieved the optimal performance value in the optimal initial cavity length or so.

#### 3.1.3. Influence of Incident Light Wavelength

**Figure 10:**
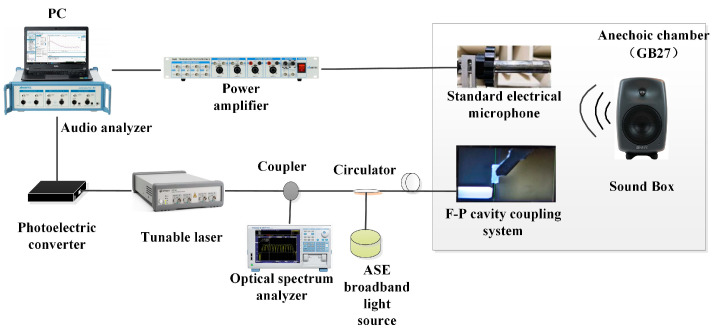
Device diagram of wavelength experiment system.

In order to determine the input to the F-P cavity of the best incident light wavelength and explore the sensor’s acoustic performance indicators with the wavelength of the change rule, a tunable filter was chosen for this experiment which achieved the purpose of splitting light by changing the wavelength of the diffracted light with a channel spacing of 0.4 nm. The device diagram of the experimental system is shown in Figure 10.

The selected wavelength range is based on the use of the FPGA demodulation board. Half a cycle can determine the best range, and in the case of the light source of the incident light wavelength of 1550.12 nm, it can determine, according to Equation (5), the wavelength range of this experiment by using a tunable filter to regulate the wavelength range of the incident on the photodetector to about 1547~1553 nm.

(5) $$\begin{array}{l} \frac{4n\pi L}{\lambda_{1}}-\frac{4n\pi L}{\lambda }=\frac{\pi }{2}\\ \frac{4n\pi L}{\lambda }-\frac{4n\pi L}{\lambda_{2}}=\frac{\pi }{2} \end{array}$$

**Figure 11:**
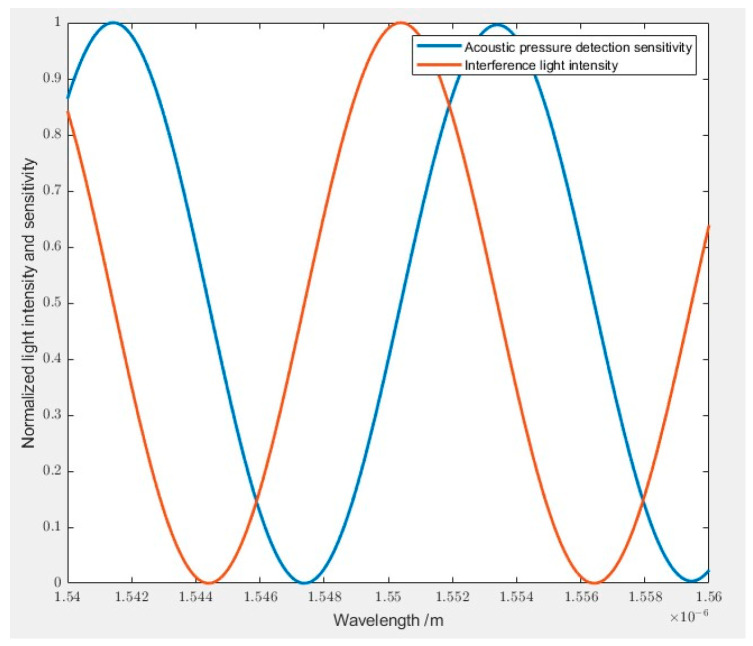
Plot of interferometric light intensity and acoustic pressure detection sensitivity versus wavelength.

According to Equations (6) and (7), the relationship between the wavelength and the interference light intensity input to the photodetector and the acoustic pressure detection sensitivity of the sensor can be drawn, as shown in Figure 11, where $R_{Fiber}\left(\lambda \right)$ and $R_{MEMS}\left(\lambda \right)$ are taken as 4%, λ is 1550.12 nm, $I_{i}(\lambda )$ is 1.5 mW, and the positive scale factor k is taken as 1.

(6) $$I_{FP}\left(\lambda \right)=\left[R_{Fiber}\left(\lambda \right)+R_{MEMS}\left(\lambda \right)+2\sqrt{R_{Fiber}\left(\lambda \right)R_{MEMS}\left(\lambda \right)}\cos\left(\frac{4\pi L}{\lambda }\right)\right]I_{i}(\lambda )$$

(7) $$S=-\frac{8\pi kI_{i}(\lambda )}{\lambda }\sqrt{R_{Fiber}\left(\lambda \right)R_{MEMS}\left(\lambda \right)}\sin(\frac{4\pi L}{\lambda })$$

**Figure 12:**
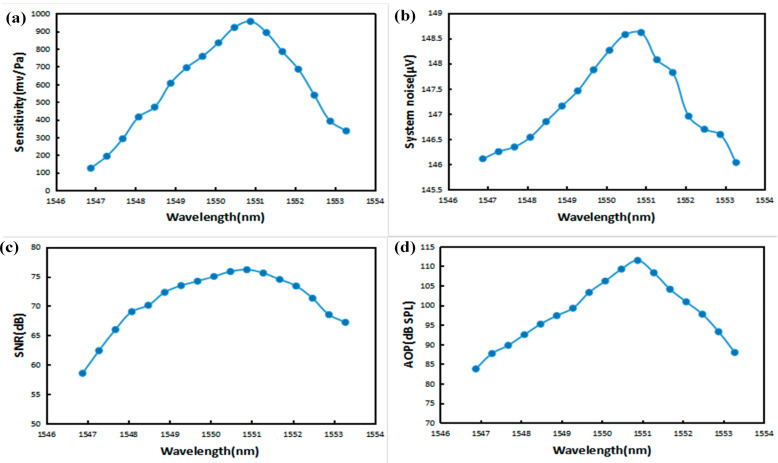
(**a**) Plot of sensitivity versus wavelength. (**b**) Plot of systematic bottom noise versus wavelength. (**c**) Plot of SNR versus wavelength. (**d**) Plot of AOP versus wavelength.

In the experiment, the cavity length of the F-P cavity coupling system was still adjusted to the optimum initial cavity length state, and the incident light power of the light source was approximately 1.5 mW. In the wavelength range of 1547~1553 nm, with the increase in the incident light wavelength, the sensitivity of the sensor, the bottom noise of the system, the SNR, and the AOP have a maximum value in this wavelength range, and the optimal input wavelength is 1550.88 nm, as shown in Figure 12.

The trend of the acoustic indicators of the sensor with the wavelength is the same as the trend of the acoustic pressure detection sensitivity and interfering light intensity with the wavelength. This is because the intensity of the interfering light input to the photodetector changes with the acoustic pressure sensitivity of the sensor, while the reflected light from the F-P cavity is detected by the photodetector (PD). The output voltage is proportional to the input light intensity, and as a result, the intensity of the optical signal, which is converted into an electrical signal by the demodulation device, shows the same trend of change. The electrical signal is finally input to the audio analyzer, and then the measured acoustic performance shows a similar trend. 

From this set of experimental results, it can be seen that, by changing the wavelength of incident light to the F-P cavity through the tunable filter, the range of optimal incident light wavelengths for which the acoustic performance index of the sensor is optimal can be quickly determined. This provides a new experimental scheme for determining the orthogonal operating Q-point in the future.

### 3.2. Sensor Fabrication and Performance Testing

**Figure 13:**
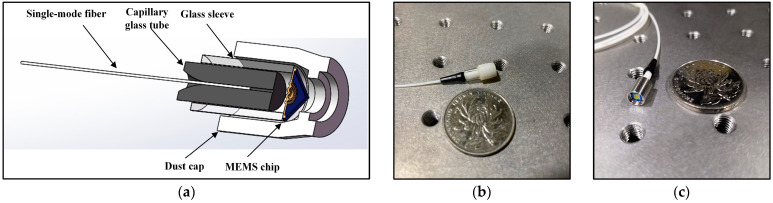
(**a**) Structure of MOEMS fiber-optic acoustic pressure sensor. (**b**) Physical image of the sensor with a 5-ring structure chip. (**c**) Physical image of the sensor with a 9-ring structure chip.

Figure 13a illustrates the schematic structure of the EFPI fiber-optic acoustic pressure sensor based on the MOEMS’s sensitive structure, which consists of four parts: a single-mode fiber core, a glass sleeve, a MOEMS chip, and a dust cap. Figure 13b shows the physical diagram of the sensor fabricated by using a 5-ring chip. Figure 13c shows the optimized sensor using a 9-ring chip.

The encapsulation process of the sensor coupling is as follows: (1) Ultraviolet adhesive is bonded to one side of the glass sleeve of the sensor chip to ensure that the centers of the two are aligned. (2) Ultraviolet light irradiation curing is glued to the chip of the glass sleeve and fixed onto the six-axis precision displacement stage. Single-mode optical fiber ceramic inserts are fixed onto the other displacement stage and aligned with the center of the chip, rotating the displacement stage to the cavity. The other side of the single-mode fiber is connected to the spectrum analyzer, the FSR reaches the theoretical value of 12 nm, the interference intensity spectral contrast reaches a maximum of about 27.9 dB, and the length of the F-P cavity is about 100 μm. (3) The F-P cavity is fixed by gluing the end of the ceramic insert to the glass sleeve. Finally, the experimentally fabricated EFPI acoustic pressure sensor with a size of 12 mm × 5 mm has a loss factor of 0.2 for the F-P cavity in the 1531 nm to 1564 nm band.

**Figure 14:**
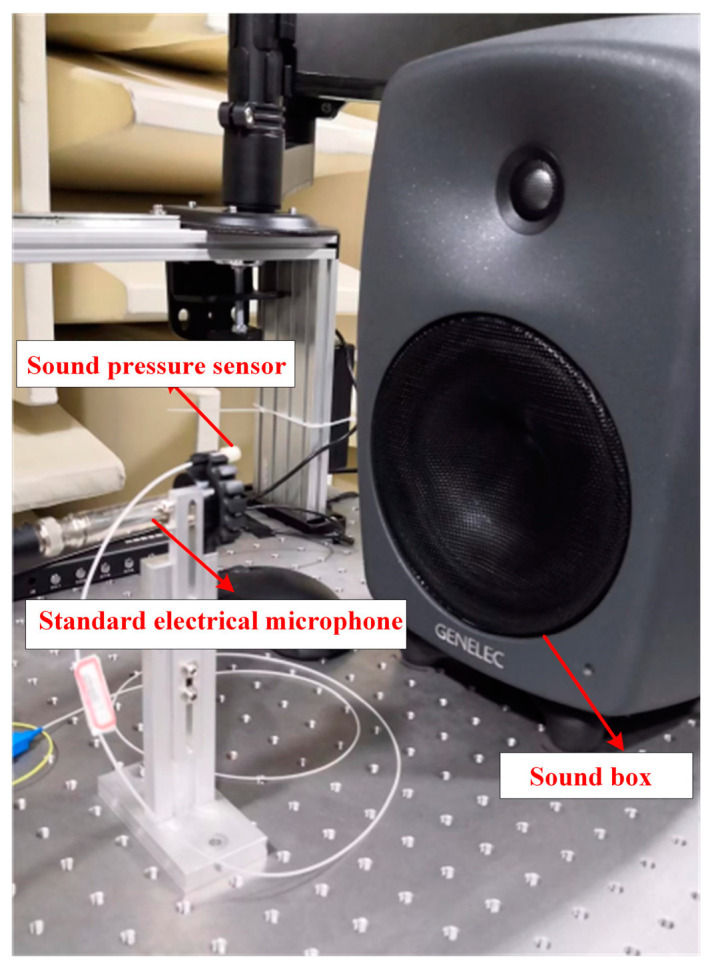
Acoustic test live view.

**Figure 15:**
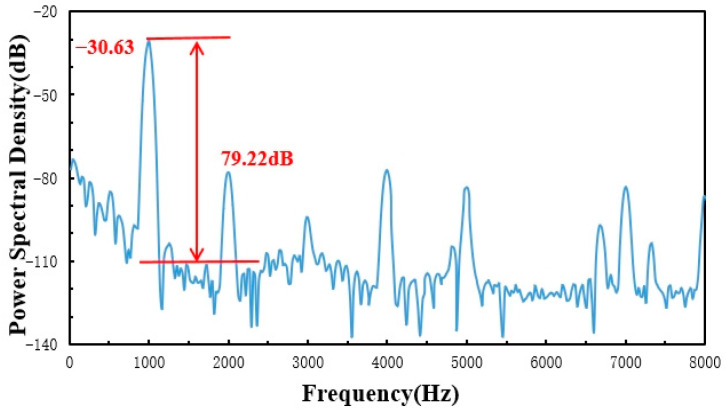
SNR test results.

**Figure 16:**
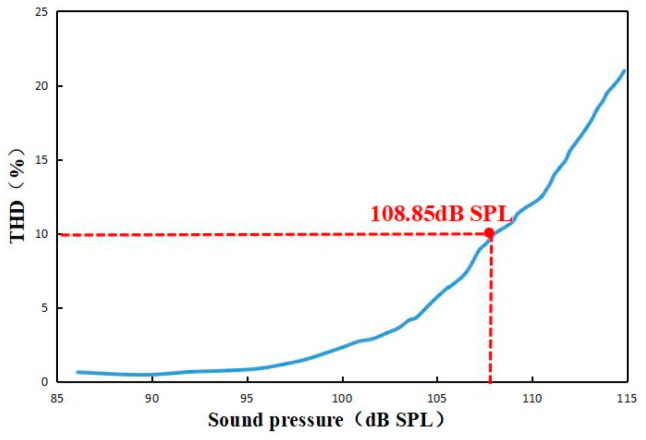
AOP test results.

**Figure 17:**
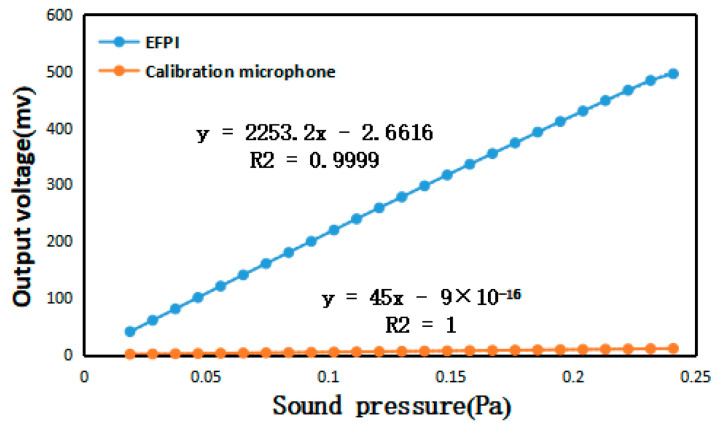
Sensitivity test results.

The optimized fabricated sensor was applied to the whole system, and a comprehensive standard acoustic test was conducted in a semi-anechoic chamber environment, as shown in Figure 14. The optical power of the light source was adjusted to 1.5 mW, and the wavelength of the incident light incident to the F-P cavity was adjusted to the optimal value of 1550.88 nm from the above experiments. The results of the acoustic indices are shown in Figure 15, Figure 16 and Figure 17.

## 4. Conclusions

We proposed and demonstrated a highly sensitive high-AOP fiber-optic acoustic pressure sensor based on a MOEMS sensitized structure. Increasing the number of sensitized rings on the acoustic thin film improved its acoustic detection sensitivity. The use of the silicon oxide thin film process and wafer-level integration technology as the means of acoustic thin film processing also greatly improved the stability and consistency of the acoustic pressure sensor. The application of a new methodology of real-time coupled acoustic testing simultaneously characterized the spectroscopic and acoustic performances of the sensor; as the F-P cavity length increased, the FSR decreased, the interferometric intensity spectral contrast decreases, and the loss in the cavity increased. The results of the actual experiments are consistent with the theoretical derivations, and they derive the optimal input parameters applicable to the system.

The experimental results show that this type of sensor has an excellent performance of high sensitivity, good sound quality, and high audio discrimination. The interference intensity spectral contrast can reach 27.9 dB, which causes the sensor to have a high sensitivity of up to 2253.2 mV/Pa, which is about 50 times that of a standard electric microphone. The SNR and AOP can reach 108.85 dB SPL and 79.22 dB, which are much higher than other fiber-optic acoustic pressure sensors of the same type.

The fabricated sensor can not only realize the detection of weak sound signals, but can subsequently be built into transformers, GIS (GAS-insulated SWITCHGEAR), and other high-voltage electrical equipment to measure the ultrasonic signals generated by partial discharges. This work provides a viable means for the batch fabrication of high-performance transducers and their practical applications in high-sensitivity, high-fidelity, terminal-passive, and long-distance bidirectional audio transmission.

## Figures and Tables

**Table 1:** Comparison of the effect of the number of sensitized rings on the performance of the sensor.

Type	F-P Cavity Loss (dBm)	Sensitivity (mV/Pa)	System Bottom Noise (μv)	AOP (dB SPL)	SNR (dB)
Non-sensitized ripple structure	18.24	576.5	167.26	94.24	70.75
3-ring sensitized ripple structure	17.53	684.2	168.32	98.96	72.18
5-ring sensitized ripple structure	17.86	886.5	169.66	102.52	74.36
9-ring sensitized ripple structure	16.68	1574.9	182.24	108.12	78.73

## Data Availability

Not applicable.
